# An item analysis according to the Rasch model of the German 12-item WHO Disability Assessment Schedule (WHODAS 2.0)

**DOI:** 10.1007/s11136-021-02872-8

**Published:** 2021-05-20

**Authors:** Lusine Vaganian, Sonja Bussmann, Maren Boecker, Michael Kusch, Hildegard Labouvie, Alexander L. Gerlach, Jan C. Cwik

**Affiliations:** 1 Department of Clinical Psychology and Psychotherapy, Faculty of Human Sciences, University of Cologne, Pohligstr. 1, 50969 Cologne, Germany; 2 Institute of Medical Psychology and Medical Sociology, University Hospital of RWTH Aachen, Aachen, Germany; 3 Department of Internal Medicine, Section: Clinical Psycho-Oncology, Working Group Psycho-Oncological Health Services Research, University Hospital of Cologne, Cologne, Germany

**Keywords:** WHODAS 2.0, Disability, Cancer, Rasch analysis, Psychometric properties

## Abstract

**Purpose:**

The World Health Organization Disability Assessent Schedule 2.0 (WHODAS 2.0) assesses disability in individuals irrespective of their health condition. Previous studies validated the usefulness of the WHODAS 2.0 using classical test theory. This study is the first investigating the psychometric properties of the 12-items WHODAS 2.0 in patients with cancer using item analysis according to the Rasch model.

**Methods:**

In total, 350 cancer patients participated in the study. Rasch analysis of the 12-items version of the WHODAS 2.0 was conducted and included testing unidimensionality, local independence, and testing for differential item functioning (DIF) with regard to age, gender, type of cancer, presence of metastases, psycho-oncological support, and duration of disease.

**Results:**

After accounting for local dependence, which was mainly found across items of the same WHODAS domain, satisfactory overall fit to the Rasch model was established (*χ*^2^ = 36.14*, p* = 0.07) with good reliability (PSI = 0.82) and unidimensionality of the scale. DIF was found for gender (testlet ‘Life activities’) and age (testlet ‘Getting around/Self-care’), but the size of DIF was not substantial.

**Conclusion:**

Overall, the analysis results according to the Rasch model support the use of the WHODAS 2.0 12-item version as a measure of disability in cancer patients.

## Introduction

About 15% of the world’s population live with some form of disability [1]. According to the World Health Organization (WHO), a person's functioning and disability are best described by a dynamic interaction between contextual factors and health conditions [2]. In addition to establishing a patient’s diagnosis, it is necessary to assess the overall condition in particular areas of life (i.e., the disability of a patient with regard to home tasks, work or other social areas) in order to ensure sound clinical decision-making and selection of appropriate interventions for patients [3]. Since disability can affect many life areas, it is difficult to ensure a suitable, reliable and valid measurement of its impact on the live of a person.

In 2001, the WHO developed the International Classification of Functioning, Disability, and Health (ICF) and defined disability as “an umbrella term for impairments, activity limitations or participation restrictions” [2]. Based on the ICF, the World Health Organization's Disability Assessment Schedule 2.0 (WHODAS 2.0) was developed to provide a standardized method for measuring health and disability [3]. The fifth edition of the Diagnostic and Statistical Manual of Mental Disorders (DSM-5) [4], recommend the WHODAS 2.0 as "the best current measure of disability for routine clinical use" [5].

The scale is an established tool for the assessment of functioning difficulties in six domains (cognition, mobility, self-care, getting along, life activities, and participation). It has been developed for individuals with any kind of disease and is available in three different length regarding the number of items (12, 12 + 24, and 36 items) and as interview-, self- or proxy-administered versions [3]. Usage of the WHODAS 2.0 is continuously increasing, and it is available in 47 languages and dialects [6]. It has been validated for different health conditions, for example, depression [7], multiple sclerosis [8], or myocardial infarction [9].

Cancer patients have to cope with their diagnosis and master the disease-associated tasks and changes. In addition, they may also suffer from disability. The disabilities experienced by cancer patients can differ substantially, due to the heterogeneity of cancer entities and individual disease progression. Thus, it is pertinent to consider the application of the WHODAS 2.0 in the oncological context as well.

Research studying the psychometric properties of the WHODAS 2.0 in an oncological context is rare. Only few studies exist based on classical test theory (CTT), which showed good to excellent reliability, good convergent and discriminant validity, and supported the 6-domain structure [10, 11]. However, within Chinese breast cancer patients a 7-domain structure was identified [12]. An advantageous alternative to CCT is item analysis according to the Rasch Model, which can be used to assess the unidimensionality of the items, sampling invariance, and local dependence problems [13, 14] According to Rasch (1965), this must be re-examined for each new population the measure is applied to [as cited in 14]. Studies employing Rasch analysis on the WHODAS 2.0 have looked at different health conditions like myocardial infarction, stroke, osteoarthritis, depression, and brain injuries [9, 15–18]. These studies confirmed the assumption of unidimensionality for the 36 item version as well as 12 items short version of the WHODAS 2.0.

However, to the best of the authors' knowledge, Rasch analysis has not yet been applied to the WHODAS 2.0 in an oncological context. That is why this study aims to examine the applicability of the 12-items version of the WHODAS 2.0 among patients afflicted by various types of cancer with the aid of Rasch analysis, especially to investigate the assumptions of unidimensionality, invariance across different exogenous variables, local independence of items, and the targeting.

## Method

### Participants and procedure

Participants were invited to participate in the study using SoSciSurvey [19] as an online survey consisting of various questionnaires. The link was posted on social media platforms and online cancer support groups as part of a validation study [20]. All participants gave their informed consent online. Inclusion criteria were: age ≥ 18 years and current or in the past cancer diagnosis. Exclusion criteria were not defined. In total, *N* = 350 cancer patients (283 women (80.9%), 66 men (18.9%), 1 gender diverse (0.3%)) completed the 12-items version of the WHODAS 2.0.

We received the permission of WHO for utilization of the WHODAS 2.0 (License: CC BY-NC-SA 3.0 IGO). All procedures contributing to this work comply with the relevant national and institutional committees' ethical standards on human experimentation and with the Helsinki Declaration of 1975, as revised in 2008. The work was approved by the Ethics Commission of the University's Faculty of Medicine (reference number 18-098).

### Assessment instruments

WHODAS 2.0. Global health status was assessed using the German version of the 12-item self-administered version of the WHODAS 2.0 [3]. The scale is an established and validated tool for the assessment of functioning difficulties in six domains (understanding and communicating, mobility, self-care, getting along, life activities, and participation). The participants estimate how many difficulties they have had in performing various activities in the last 30 days on a 5-point Likert-type scale (none = 0, mild, moderate, severe, extreme/cannot do = 4). Higher scores reflect a more significant disability [3].

Statistical analyses.

Data were analyzed using SPSS version 26.0 [21] and RUMM2030 software [22]. Patients’ characteristics are described by means and standard deviations. One item is missing from one patient of the 12-item WHODAS version, which was replaced by using the mean of the other items as recommended by Üstün et al. [3].

Item analysis methodology according to the Rasch model was used to assess the psychometric properties of the WHODAS 2.0 in an oncological context. This model allows a nuanced analysis of an instrument's psychometric properties because it focusses on the items and how persons respond to them. Person parameters are estimated, which express the individual extent of a latent trait, which in the case of WHODAS 2.0 is disability [23]. Likewise, on the same latent trait, the item difficulty parameters are estimated. ‘Easy’ WHODAS-items would be items that are already scored high in the direction of disability by patients with only minor disabilities, whereas ‘difficult’ WHODAS-items would be items that are only scored high by patients with major disabilities. During the process of the item analysis according to the Rasch model, it is tested whether patients respond as expected to each item. For example, a patient with major disabilities should also score high on an ‘easy’ WHODAS-item. In order to properly test the fit of the WHODAS-data to the Rasch model, this paper follows the current state-of-the-art Rasch analysis requirements [24] and the CREATE guidelines for reporting valuation studies [25].

Given the polytomous WHODAS-items, the Partial Credit Model (PCM) [26] was used. According to the Rasch model, performing analysis comprises the investigation of how well the data meet the expectations of the measurement model, i.e., unidimensionality, local independence, and absence of differential item functioning (DIF). In this sense, the analysis according to the Rasch model can be understood as an iterative process in which potential deviations from the model’s expectations are investigated and—if possible—resolved.

One fundamental requirement of the Rasch model is unidimensionality, i.e., the items of a scale should assess only one underlying construct. Unidimensionality was tested with principal component analysis (PCA) of the residuals [27]. The idea is to use the items with the highest negative/positive loadings on the first component to create two subsets of items. The separate person estimates of these two subsets are used to identify significant differences using independent *t* tests. The proportion of significant *t*-tests should not exceed 5% to confirm unidimensionality [28].

Another assumption is that of local independence. This implies that there should be no residual correlations between items when extracting the trait factor [24]. Locally dependent items, respectively, items which are linked in some way, can lead to overestimation of reliability, parameter estimation bias, and problems with construct validity [29]. Following the recommendations of Christensen et al. [29] and Marais [30], a cut-off value of 0.2 above the average residual correlation was used to assess local dependence (LD). One strategy to deal with LD if one does not want to delete scale items is to combine the locally dependent items into testlets by adding them together. Using the testlet-strategy results in a bi-factor equivalent solution. The proportion of explained common variance (ECV) [31–33] of the general factor should be > 0.9 to consider the scale as unidimensional. The ECV is indicated in RUMM2030 as A-factor [31]. One more assumption is that there is no item bias with regard to exogenous variables (no DIF). If DIF is given, the difficulty of an item is different for different groups (e.g., males and females). In other words, in different groups, the corresponding item indicates the latent characteristic in different ways [24]. DIF analyses were examined using analysis of variance (ANOVA). We tested the items for DIF by looking at gender (woman, man), age (median split of the sample: below and above 54), type of cancer (breast, other forms of cancer, multiple cancers), presence of metastases (yes, no, unknown), psycho-oncological support (yes, no) and duration of disease (median split of the sample: below and above 3.9 years). In case of DIF, we evaluated the impact of DIF by computing equated scores [34]. Due to too small group sizes, we had to exclude the one gender diverse person for the DIF analysis of gender and combine the residual cancer types into one category, 'other forms of cancer' for the DIF analysis of cancer type.

Additionally, item fit as indicated by standardized residuals within a range of ± 2.5 and overall model fit indicated by a non-significant Chi-Square probability *p* > 0.01, were investigated [27, 35]. Moreover, the ordering of item thresholds was analyzed. Item thresholds are the transition points between two adjacent respond categories. Disordered thresholds may affect scale scores’ interpretation and validity [36]. There can be different causes for threshold disordering, such as that the respondents might have difficulties consistently differentiate between the different response options, or LD might cause the disordering. If the disordering is due to category differentiation problems, one way to handle this is by collapsing the disordered response categories.

The scale's internal consistency was estimated using Person Separation Index (PSI). The PSI is equivalent to Cronbach's alpha and can be interpreted similarly with a requirement of a minimum value of 0.7 for group and 0.85 for individual use [24]. Targeting was assessed graphically based on the person-item threshold distribution graph. Person-item maps demonstrate how person parameters and item thresholds are distributed along the trait dimension.

## Results

Mean age of the *N* = 350 participants was 52.34 years (SD = 14.07) and all participants completed the WHODAS 2.0 questionnaire. A selection of descriptive statistics and an overview of cancer diagnoses among the participants are presented in Table 1.

**Table 1:** Characteristics of cancer patients (*N* = 350)

Gender	
Male	66 (18.9)
Female	283 (80.9)
Divers	1 (0.3)
Age (in years)	52.34 ± 14.07 (20–83)
Job situation	
Active	146 (41.7)
Certified sick	56 (16.0)
Different form	148 (42.3)
Types of cancer	
Breast	182 (52.0)
Urological	37 (10.6)
Prostate, testicular	33 (9.4)
Gynecological	29 (8.3)
Hematological	26 (7.4)
Intestinal, rectal	20 (5.7)
Skin	13 (3.7)
Lungs, bronchia	10 (2.9)
Ear, nose, throat	7 (2.0)
Gastric, esophageal, pancreatic	7 (2.0)
Parts of central nervous system	5 (1.4)
Soft tissue	3 (0.9)
Residual category (including other forms of cancer)	29 (8.3)
Metastases	
No	260 (74.3)
Yes	78 (22.3)
Unknown	12 (3.4)
Current psycho-oncological, psychological, psychotherapeutic support	
No	251 (71.7)
Yes	99 (28.3)
HADS-T Score—Distress (HADS-T ≥ 15)	154 (44.0%)

Values are presented in frequency (%) or mean±standard deviation (range)

*HADS-T* Hospital Anxiety Depression Scale [38]

(To identify patients with an increased need for psycho-oncological care and especially for depression symptoms in cancer patients, a sum score of HADS-T ≥ 15 can be used as the cut-off value) [39]

The initial analysis of all 12 items of the WHODAS 2.0 showed a satisfactory overall model fit (*χ*^2^ = 88.21, *p* = 0.01). However, several items displayed LD, two items showed item misfit, DIF was found for items 1 and 12 in relation to age, for items 7 and 11 in relation to gender, and for item 12 related to disease duration and disordered thresholds in six items. In the initial analysis, LD was found for item 1/2/7, item 3/6, item 7/8, item 8/9, and 10/11.

As LD seemed to be the major problem, we focused at first on accounting for it. We stepwise combined the locally dependent items with the highest residual correlation, starting with the item pair 8 and 9 (*r* = 0.554; critical-LD value = 0.1). These successively combined locally dependent items were consistent with the six WHODAS 2.0 domains (in order of testlet formation: 'Self-care', 'Getting along with people', 'Getting around', 'Understanding and Communicating'', Life activities' and 'Participating in society'). After combining the two items of each domain into one testlet, LD was still present between the domain testlets 'Getting around' and 'Self-care' (*r* = 0.102; critical LDvalue = 0.1), which were subsequently combined to one common testlet. The fit statistics of the testlets of the WHODAS 2.0 [3] can be found in Table 2.

**Table 2:** Final analysis fit statistic of the WHODAS 2.0 12-items version (testlets ordered by location)

Testlet	Item	Location	SE	Residual
Participating in society	4) How much of a problem did you have joining in community activities (for example, festivities, religious or other activities) in the same way as anyone else can? 5) How much have you been emotionally affected by your health problems?	− 0.64	0.04	− 0.72
Life activities	2) Taking care of your household responsibilities? 12) Your day to day work?	− 0.25	0.04	− 2.09
Getting around/ Self-care	1) Standing for long periods such as 30 min? 7) Walking a long distance such as a kilometer (or equivalent)? 8) Washing your whole body? 9) Getting dressed?	0.23	0.03	0.18
Understanding and communication	3) Learning a new task, for example, learning how to get to a new place? 6) Concentrating on doing something for ten minutes?	0.28	0.04	0.17
Getting along with people	10) Dealing with people you don’t know? 11) Maintaining a friendship?	0.39	0.04	1.31

*SE* Standard error

After applying these strategies, there was no further evidence of LD nor item misfit. The assumption of unidimensionality could be confirmed. The *t*-test showed satisfactory results with 11 significant tests (3.30%). The A-factor was 0.94, indicating a high explained common variance across the five testlets and confirming the scale’s unidimensionality as well.

However, in the final analysis DIF related to age was found for the testlet ‘Getting around/ Self-care’ and related to gender for testlet ‘Life activities'. Elderly persons seemed to have more difficulties in the domain 'Getting around/ Self-care' than younger persons with the same level of disability, and women seemed to have more difficulties in ‘Life activities’ than men with the same level. We investigated the impact of found DIF with the before mentioned methods. After splitting the testlet ‘Life activities’ for gender-DIF and computing equated scores, only a minor difference was found, with the biggest difference being 1.5 score points. As the gender-DIF was considered as being not substantial, we decided not to split this testlet for gender in the final solution. The situation was similar regarding the age-DIF, although the difference in equated scores between the younger and older patients was slightly higher, with a maximum score difference of about 2 points in the middle range of the person location (between 0 and 1). However, in the other parts of the disability dimension, the difference was negligible. Additionally, we conducted an analysis to examine the impact of the found age-DIF in the present sample: Mean WHODAS 2.0-person parameters between the younger and older patients once with and once without adjusting for DIF were compared. The effect size [37] for the comparison of younger and older patients without DIF adjustment was *d* = 0.44, whereas it was *d* = 0.52 with DIF adjustment. Based on only minor differences in both lines of analyses, we decided not to split for age in the final solution.

After adjusting for LD, two testlets displayed disordered thresholds: the testlet ‘Understanding and communicating’ showed negligible disordering in the first two thresholds: Threshold 1 = − 0.61; Threshold 2 = − 0.63. The other thresholds in this testlet were ordered. The testlet ‘Getting along with people’ (item 10 and 11) showed more disordering. Several lines of additional analyses were performed, e.g., collapsing for the initial item 11, which had displayed disordering in the initial analysis as well, or rescoring items 10 and 11. However, disordering for the testlet ‘Getting along with people’ still remained, and model fit did not improve. For this reason, and the reason that the final solution with five testlets (without rescoring) met the expectations of the measurement model, we did not make any further optimization regarding threshold ordering.

The overall model fit of the final solution was satisfactory (*χ*^*2*^ = 88.21, *p* = 0.07) with good reliability PSI = 0.82. Table 3 shows the summary fit statistic of the initial analysis, as well as of the analysis with the six domains and of the final analysis.

**Table 3:** Summary fit statistic

	Overall item–trait interaction			Unidimensionality *t* test	Reliability	Targeting				Item misfit	Differential Item Functioning (DIF)	Disordered thresholds
						Item Residual		Person Residual				
Analysis	Chi-square	df	*p* value	Test (%)	PSI	Mean	SD	Mean	SD	Item number	Item number (source of DIF)	Item number
Initial	88.21	60	0.01	5.41	0.87	− 0.37	1.63	− 0.29	0.95	2, 9	Age: 1, 12 Gender: 7, 11 Disease duration: 12	1, 7, 8, 9, 11, 12
Testlets (6 WHODAS 2.0 Domains)	38.17	30	0.15	4.80	0.82	− 0.51	1.40	− 0.29	0.80	None	Age: Testlet “Getting around”, “Life activities” Gender: Testlet “Life activities”	Testlet “Getting around”, “Understanding and Communicating”, “Self-Care”, “Getting along with people”
Final	36.14	25	0.07	3.30	0.82	− 0.23	1.26	− 0.31	0.96	None	Age#: Testlet “Getting around/ Self-Care”, “Life activities” Gender#: “Life activities	Testlet “Understanding and communicating”, “Getting along with people”

*DF* degrees of freedom, *PSI* person separation index, *SD* standard deviation

**#**It was not adjusted for the found DIF (see text for more details)

**Fig. 1:**
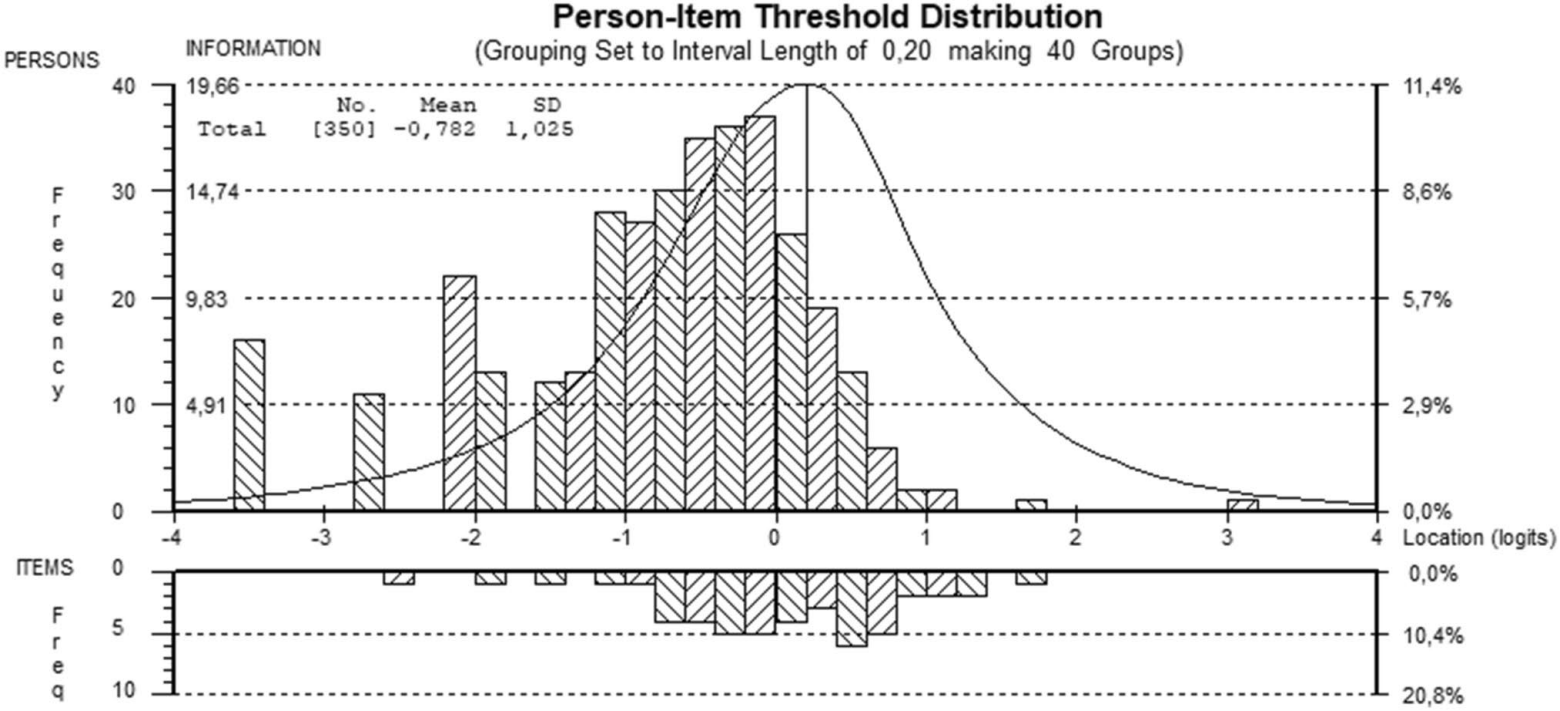
Person-Item threshold distribution (final analysis). On the top half of the graph, the distributions of persons and at the bottom half the item thresholds are shown for the final analysis of the WHODAS 2.0 12-item version with higher values indicating higher level of disability (top of the half) and higher difficulty (bottom half). At the left side, the frequency and at the right side, the percentage of persons, respectively, items are shown

Figure 1 shows the targeting of the scale with a mean person location value of *M* = − 0.78 (SD = 1.03). This result means that the patients had a lower mean level of disability than the average difficulty of the scale (which is 0). The person distribution was slightly off-centered, with more people showing lower levels of disability and only a relatively small number of persons with high levels of disability. The item threshold distribution shows that the scale measures a wide range of disability, except for very low levels and very high levels of disability.

A transformation table of the WHODAS 2.0 scores to interval-level person parameters is provided in Table 4.

**Table 4:** Conversion table of Rasch logits

WHODAS 2.0 Score	Interval-scaled person estimate
0	− 3.49
1	− 2.68
2	− 2.15
3	− 1.80
4	− 1.55
5	− 1.35
6	− 1.19
7	− 1.06
8	− 0.94
9	− 0.84
10	− 0.75
11	− 0.67
12	− 0.59
13	− 0.52
14	− 0.45
15	− 0.39
16	− 0.33
17	− 0.27
18	− 0.22
19	− 0.16
20	− 0.11
21	− 0.06
22	− 0.01
23	0.04
24	0.09
25	0.14
26	0.19
27	0.24
28	0.29
29	0.34
30	0.39
31	0.44
32	0.49
33	0.54
34	0.60
35	0.65
36	0.71
37	0.78
38	0.85
39	0.93
40	1.01
41	1.12
42	1.23
43	1.37
44	1.53
45	1.74
46	2.01
47	2.43
48	3.08

## Discussion

This study aimed at assessing and is the first to provide information about the psychometric properties of the 12-item version of the WHODAS 2.0 within a sample of cancer patients using modern psychometric analysis, i.e., Rasch analysis. The use of Rasch analysis has numerous potential advantages over CTT when assessing self-reported health outcomes. For example, it allows a nuanced analysis of the psychometric properties because of its focus on single items and how persons respond to them, it permits testing bias or DIF in different subgroups, and facilitates a transformation of ordinal into interval-level scores. The use of the interesting and cancer-specific DIF variables should be highlighted. Overall, the Rasch measurement model's application on the WHODAS 2.0 showed a good model fit with good reliability after making some modifications related to LD.

The scale showed several pairs of locally dependent items corresponding to the domains of the WHODAS 2.0 [3]. After combining the locally dependent item pairs successively into domain-specific testlets, one last LD could be observed between the testlets 'Getting around' and 'Self-care', which had to be combined to one common testlet. In terms of content, this makes sense since both assess facets of activities of daily living (ADL). The findings of LD within the scale are comparable with other studies. For example, Luciano et al. [7] reported correlated pairs of items within the domains ‘Getting around’, ‘Self-care’ and ‘Getting along with people’ or Snell et al. [16] within the domain 'Self-care'. We found LD in all domains of the WHODAS 2.0 like Kutlay et al. [17] or Küçükdeveci et al. [18] and additionally one between the two domains assessing ADL.

DIF was tested by gender, age, type of cancer, the presence of metastases, psycho-oncological support, and duration of disease. For most of these external variables, no DIF was found. However, in contrast to other studies [e.g., 9], uniform DIF occurred related to age for testlet ‘Getting around/ Self-care’ and related to gender for testlet ‘Life activities'. After investigating the impact of the found DIF with splitting for gender and computing equated scores, we only found a relatively small inconsiderably difference in the equated scores, so we decided not to split for gender. However, there was a bigger difference with a maximum score difference of about 2 points in the middle range of the person's location regarding age. This result denotes that patients with the same level of disability responded differently to the items of the ADL-testlet dependent on their age. Specifically, elderly individuals seem to have more difficulties in this domain than younger persons with the same level of disability. However, this difference becomes visible only in the middle range. In contrast, patients with either a high or low level of disabilities responded comparable in the areas of high or low level of disability, irrespective of their age. Another consideration about the found minor DIF might be that this is not a measurement bias, but the difference could be expected. People develop indeed more difficulties with higher age in areas of ‘Getting around’ and ‘Self-care’, so a split for age would not be necessary. Given that the DIF was found only in a tiny part of the assessed dimension and given the only minor differences (in term of effect sizes) when comparing younger and older patients with and without the DIF adjustment as well as the contentual reflection, about expected differences, we decided not to adjust for DIF. However, our sample is relatively young, with a mean age of 52.34 years. In a sample with more elderly patients, a more relevant age-DIF might be found.

The confirmation of unidimensionality of the scale is consistent with other Rasch analyses on the WHODAS 2.0 [9, 15]. Additionally, targeting (Fig. 1) was satisfactory for the present sample with a mean person location value of *M* = -0.78 (SD = 1.03). However, for low and high levels of disability, the targeting is not as good as item thresholds are missing in these areas of the dimension. The WHODAS 2.0 was initially developed to provide a standardized method for measuring health and disability in the general population [3]. Our results indicate that even in a sample of patients with cancer, the differentiation in the lower segment of disability is not optimal—an area where probably most of the people of a healthy population would be located. However, the differentiation within a healthy population or persons with no, respectively, very low levels of disability may not be so relevant for assessment of oncology patients and the improvement of clinical decision-making in psycho-oncology. However, more difficult items are also missing, making it hard to precisely assess disability in patients with a high level of disability using the 12-items version of the WHODAS 2.0. A good example is the *Getting around*-domain. In the 12-item version, the items "Standing for long periods such as 30 min?" and "Walking a long distance such as a kilometer (or equivalent)?" are indicators for this domain activities that might be far too difficult to perform for severely ill patients. Here it might be sensible to either include some more items of the WHODAS 2.0 36 items version or develop a better targeted short scale for patients with a higher level of disability (e.g., with WHODAS-items like: "Moving around inside your home.").

In the initial analysis of our study, disordered thresholds were found for six items. In the testlet solution, less disordering was found, indicating that at least part of the threshold disordering in the initial analysis was due to LD. However, the testlet “Getting along with people” displayed disordered thresholds, a phenomenon often observed for testlets. Therefore, the ordering of thresholds should be further investigated in future WHODAS studies.

Besides some strengths, the present study also has limitations. There is a relatively high percentage of breast cancer patients in the sample of this study. Accordingly, the results may only be generalized to cancer patients with caution. Due to small group sizes, we had to combine the residual cancer types into one category, ‘other forms of cancer', for DIF analysis. To examine the influence of various cancer forms decidedly, especially cancer types with more severe disease progress, additional research would be interesting and important. Nevertheless, in our study, we could use the presence of metastases or the duration of disease as an indicator for the severity. Both of these indicators showed no DIF. Also, the sample's psychological distress, measured by the HADS-T, is roughly equally distributed across the cancer forms. We therefore can assume that the type of cancer does not unduly influence the response behavior. Furthermore, the sample was recruited from social media platforms and within online cancer support groups. As a result of this, the sample is relatively young, with a mean age of 52.34 years. The scale's targeting was good for the present sample but already shows an off-centered person distribution with a relatively small frequency of persons with a high level of disability. This result indicates a bias by low disability levels in this sample. Also, a high percentage (41.7%) of the cancer patients have an active job situation, indicating a relative fit sample. The item threshold distribution shows that the scale measures a wide range of disability but not across the entire range. With respect to this and the small age-DIF we found in our study, future research should examine a sample with a higher level of disability and perhaps include some additional items suited for the assessment of higher levels of disability.

## Conclusion

The present study provides essential information about the psychometric properties of the 12-items version of the WHODAS 2.0 in the oncological context. The Rasch analysis of the 12-items version of the WHODAS 2.0 showed that this measurement may be used well in the oncological context, especially those who have an impairment are adequately assessed with it. The instrument is non-biased with respect to gender, type of cancer, the presence of metastases, psycho-oncological support, and duration of disease. There might be only a need for critical consideration with respect to age, especially in the elderly.

## Data Availability

Data not published within the article are available after approval by a regional ethical review board and can be shared by reasonable request.

## Code availability

SPSS code can be shared upon reasonable request.

## References

[1] World Health Organization [WHO], & The World Bank (2011). World report on Disability.

[2] World Health Organization (2001). International classification of functioning, disability and health.

[3] Üstün TB, Kostanjesek N, Chatterji S, Rehm J (2010). Measuring health and disability: Manual for WHO Disability Assessment Schedule (WHODAS 2.0).

[4] American Psychiatric Association (2013). Diagnostic and statistical manual of mental disorder: DSM-5™.

[5] Gold LH (2014). DSM-5 and the assessment of functioning: The World Health Organization Disability Assessment Schedule 2.0 (WHODAS 2.0). Journal of the American Academy of Psychiatry and the Law Online.

[6] Federici S, Bracalenti M, Meloni F, Luciano JV (2017). World Health Organization disability assessment schedule 2.0: An international systematic review. Disability and Rehabilitation.

[7] Luciano JV, Ayuso-Mateos JL, Fernández A, Serrano-Blanco A, Roca M, Haro JM (2010). Psychometric properties of the twelve item World Health Organization Disability Assessment Schedule II (WHO-DAS II) in Spanish primary care patients with a first major depressive episode. Journal of Affective Disorders.

[8] Magistrale G, Pisani V, Argento O, Incerti C, Bozzali M, Cadavid D (2015). Validation of the World Health Organization Disability Assessment Schedule II (WHODAS-II) in patients with multiple sclerosis. Multiple Sclerosis Journal.

[9] Kirchberger I, Braitmayer K, Coenen M, Oberhauser C, Meisinger C (2014). Feasibility and psychometric properties of the German 12-item WHO Disability Assessment Schedule (WHODAS 2.0) in a population-based sample of patients with myocardial infarction from the MONICA/KORA myocardial infarction registry. Population Health Metrics.

[10] Norouzi H, Roohi S, Shahhosseini M, Nouri Ghasemabady R (2020). Psychometric properties of the World Health Organization Disability Assessment Scale 2.0 among Iranian Cancer Patients. Middle East Journal of Cancer.

[11] Pösl M, Cieza A, Stucki G (2007). Psychometric properties of the WHODASII in rehabilitation patients. Quality of Life Research.

[12] Zhao HP, Liu Y, Li HL, Ma L, Zhang YJ, Wang J (2013). Activity limitation and participation restrictions of breast cancer patients receiving chemotherapy: psychometric properties and validation of the Chinese version of the WHODAS 2.0. Quality of Life Research.

[13] Fischer GH (1987). Applying the principles of specific objectivity and of generalizability to the measurement of change. Psychometrika.

[14] Gustafsson J-E (1980). Testing and obtaining fit of data to the Rasch model. British Journal of Mathematical and Statistical Psychology.

[15] Luciano JV, Ayuso-Mateos JL, Aguado J, Fernandez A, Serrano-Blanco A, Roca M (2010). The 12-item World Health Organization Disability Assessment Schedule II (WHO-DAS II): A nonparametric item response analysis. BMC Medical Research Methodology.

[16] Snell DL, Siegert RJ, Silverberg ND (2020). Rasch analysis of the World Health Organization Disability Assessment Schedule 2.0 in a mild traumatic brain injury sample. Brain Injury.

[17] Kutlay S, Küçükdeveci AA, Elhan AH, Oztuna D, Koç N, Tennant A (2011). Validation of the World Health Organization disability assessment schedule II (WHODAS-II) in patients with osteoarthritis. Rheumatology International.

[18] Küçükdeveci AA, Kutlay Ş, Yıldızlar D, Öztuna D, Elhan AH, Tennant A (2013). The reliability and validity of the World Health Organization Disability Assessment Schedule (WHODAS-II) in stroke. Disability and Rehabilitation.

[19] Leiner, D. J. (2014). SoSci Survey (Version 2.4.00-i) [Computer Software]. Retrieved from https://www.soscisurvey.de.

[20] Cwik JC, Vaganian L, Bussmann S, Labouvie H, Houwaart S, Gerlach AL (2021). Assessment of coping with cancer-related burdens: psychometric properties of the Cognitive-Emotional Coping with Cancer scale and the German Mini-mental Adjustment to Cancer scale. Journal of Psychosocial Oncology Research and Practice.

[21] IBM Corporation (2019). IBM SPSS Statistics for Windows, Version 26.0.

[22] Andrich D, Sheridan B, Luo G (2010). RUMM 2030 [Computer software].

[23] da Rocha NS, Chachamovich E, de Almeida Fleck MP, Tennant A (2013). An introduction to Rasch analysis for psychiatric practice and research. Journal of Psychiatric Research.

[24] Tennant A, Conaghan PG (2007). The Rasch measurement model in rheumatology: what is it and why use it? When should it be applied, and what should one look for in a Rasch paper?. Arthritis and Rheumatism.

[25] Xie F, Pickard AS, Krabbe PF, Revicki D, Viney R, Devlin N, Feeny D (2015). A Checklist for Reporting Valuation Studies of Multi-Attribute Utility-Based Instruments (CREATE). PharmacoEconomics.

[26] Masters GN (1982). A Rasch model for partial credit scoring. Psychometrika.

[27] Pallant JF, Tennant A (2007). An introduction to the Rasch measurement model: An example using the Hospital Anxiety and Depression Scale (HADS). British Journal of Clinical Psychology.

[28] Smith EV (2002). Detecting and evaluating the impact of multidimensionality using item fit statistics and principal component analysis of residuals. Journal of Applied Measurement.

[29] Christensen KB, Makransky G, Horton M (2017). Critical values for Yen’s Q3: Identification of local dependence in the Rasch model using residual correlations. Applied Psychological Measurement.

[30] Marais I, Christensen KB, Kreiner S, Mesbah M (2013). Local dependence. Rasch models in health.

[31] Pomeroy IM, Tennant A, Mills RJ, Young CA, Group, T. O. S. (2020). The WHOQOL-BREF: a modern psychometric evaluation of its internal construct validity in people with multiple sclerosis. Quality of Life Research.

[32] Andrich D (2016). Components of variance of scales with a bifactor subscale structure from two calculations of α. Educational Measurement: Issues and Practice.

[33] Rodriguez A, Reise SP, Haviland MG (2016). Applying Bifactor Statistical Indices in the Evaluation of Psychological Measures. Journal of Personality Assessment.

[34] Christensen KB, Thorborg K, Hölmich P, Clausen MB (2019). Rasch validation of the Danish version of the shoulder pain and disability index (SPADI) in patients with rotator cuff-related disorders. Quality of Life Research.

[35] Siegert RJ, Tennant A, Turner-Stokes L (2010). Rasch analysis of the Beck Depression Inventory-II in a neurological rehabilitation sample. Disability and Rehabilitation.

[36] Andrich D (2013). An expanded derivation of the threshold structure of the Polytomous Rasch Model that dispels any “Threshold Disorder Controversy”. Educational and Psychological Measurement.

[37] Cohen J (1988). Statistical power analysis for the behavioral sciences.

[38] Herrmann-Lingen C, Buss U, Snaith PR (2011). Hospital Anxiety and Depression Scale-Deutsche Version (HADS-D).

[39] Jenniches I, Lemmen C, Cwik JC, Kusch M, Labouvie H, Scholten N (2020). Evaluation of a complex integrated, cross-sectoral psycho-oncological care program (isPO): A mixed-methods study protocol. British Medical Journal Open.

